# Analgesia for pain control during extracorporeal shock wave lithotripsy: Current status

**DOI:** 10.4103/0970-1591.40607

**Published:** 2008

**Authors:** Narmada P. Gupta, Anup Kumar

**Affiliations:** Department of Urology, All India Institute of Medical Sciences, New Delhi, India

**Keywords:** Analgesia, ESWL, urolithiasis

## Abstract

**Purpose of Review:**

A cooperative patient is essential in maintaining stone targeting for optimal fragmentation during extracorporeal shock wave lithotripsy (ESWL). Therefore, it is important to choose an appropriate analgesic with minimal adverse effects. The guidelines for pain management during ESWL have not been established.

**Current Status:**

Various analgesic agents including opioids (morphine, pethidine, and fentanyl), nonsteroidal anti-inflammatory drugs (NSAIDS - diclofenac, propofol, ketorolac, and piroxicam), local anesthetic agents and a number of combinations have been used during ESWL by various techniques (general anesthesia, regional anesthesia, subcutaneous and intravenous injections, patient-controlled analgesia, and monitored anesthesia care). Cutaneous creams like eutectic mixture of local anesthesia (EMLA) whether used alone or in combination with oral NSAIDS have also been used and are able to reduce analgesic requirements. Topical application of a combination of dimethyl sulfoxide and lidocaine has also been found to be effective.

**Conclusion:**

The ideal analgesic, offering optimal pain control, minimal side effects, and cost-effectiveness is still elusive. Opioids administered using various techniques, provide effective analgesia, but require active monitoring of patient for potential adverse effects. Combination therapy (oral NSAID and occlusive dressing of EMLA, DMSO with lidocaine) offers an effective alternative mode for achieving analgesia with minimal morbidity. This therapy avoids the need for general anesthesia, injectable analgesics, and opioids along with their side effects.

## INTRODUCTION

Treatment of urolithiasis has been revolutionized with the introduction of extracorporeal shock wave lithotripsy (ESWL) due to its simplicity, noninvasive nature, efficacy, and minimal morbidity.[1,2] Pain experienced during ESWL is considered to be multifactorial including type of lithotriptor used, frequency, voltage, age, and sex of patient.[3] Recent developments have made ESWL more effective, with minimal morbidity, making it possible to perform ESWL in an outpatient setting without the need for general or spinal anesthesia.[4,5] Though avoidance of general anesthesia is beneficial to patients, there is a significant concern regarding jeopardizing treatment outcomes due to use of less potent analgesic methods.[6] Analgesics commonly used during ESWL include opioids, sedative hypnotics, nonsteroidal anti-inflammatory drugs (NSAIDS), and local anesthetic creams such as EMLA.[2,6,7] Although opioids provide efficacious analgesia, they are associated with significant complications - respiratory depression, bradycardia, hypotension, nausea, vomiting, and prolonged recovery time.[8] A relaxed, cooperative patient during treatment is paramount in maintaining stone targeting for optimal fragmentation. Therefore, it is essential to choose an appropriate analgesic with minimal adverse effects. Despite reports of various studies comparing different analgesic techniques during ESWL,[7,9,10] guidelines for pain management during the procedure are not established. Pain management is usually undertaken by the urologists on the basis of their own experience often resulting in a ‘hit or miss practice’. We performed a Medline and Pubmed search (using key words ‘analgesia in ESWL’) to review the current status of premedication and intraprocedural analgesia used for achieving pain control during ESWL.

## HISTORICAL ASPECTS

The ESWL has undergone significant developments since its first description by Chaussy.[11] The approach to anesthesia for lithotripsy has considerably changed since clinical ESWL began in 1980. General anesthesia was almost always necessary in the original HM-3 lithotripter (Dornier Medtech, Germany) due to a powerful shockwave and treatment at high-energy levels causing intolerable pain. Newer version machines such as the Dornier delta-compact lithotriptor have made possible the use of less potent analgesia and sedation because of modifications in the aperture size of the shock wave source and their lower shockwave output energies. These machines have fewer complications but have lower efficacy than the original HM-3 lithotripter.[11–12,14] Though poorer treatment outcomes may be due to various factors like power source, coupling mechanism, focal zone size, and peak pressure, the potential role of inadequate analgesia during ESWL has not been fully assessed. While treatment with the third generation piezoelectric lithotriptors has been described as painless,[15] 28% patients experienced severe pain when undergoing treatment without anesthesia.[16] The most appropriate analgesia, which offers pain-free treatment, minimal side effects, and cost-effectiveness, remains to be established.

## PATHOGENESIS OF PAIN DURING ESWL

The pathogenesis of pain in ESWL is still poorly understood but is considered to be multifactorial. The cutaneous superficial skin nociceptors and visceral nociceptors such as periosteal, pleural, peritoneal, and/or musculoskeletal pain receptors are two important components responsible for causing pain during ESWL.[17] Patient-related factors and several physical variables including the type of lithotriptor, size and site of stone burden, location of the shockwave front, cavitation effects, shockwave peak pressure, size of focal zone, and area of shockwave entry at the skin are additionally responsible for pain.[9] The role of analgesia is limited to allowing application of shockwaves with appropriate timing and strength to obtain good fragmentation.

## CURRENT STATUS OF VARIOUS ANALGESIC AGENTS IN ESWL

Different analgesic agents including opioids (morphine, pethidine, and fentanyl), NSAIDS (diclofenac, propofol, ketorolac, and piroxicam), local anesthetic agents, and a number of combinations have been used during ESWL by various analgesic techniques (general anesthesia, regional anesthesia, subcutaneous and intravenous (IV) injections, patient-controlled analgesia, monitored anesthesia care [MAC], cutaneous cream).[18–20] Fentanyl, a strong synthetic narcotic, in the opioid group, is used commonly during ESWL. The fentanyl-propofol combination has been proven as an effective IV analgesic option, but has significant adverse effects like centrally mediated respiratory depression along with decrease in oxygen saturation, nausea, vomiting, drowsiness, and hypersensitivity reactions.[8,19] Therefore, regular oxygen saturation measurement is necessary, especially when this drug is used along with sedatives in ESWL. Newer opioids like remifentanil, sufentanil have been used in ESWL in the form of MAC[21–24] and regional anesthesia (intrathecal/subarachnoid route).[25–28] Remifentanil has a short elimination half-life and a rapid analgesic action. While both remifentanil and sufentanil have been found to be of equal efficacy with regards to analgesia, patient's and surgeon's satisfaction during ESWL, remifentanil has a better side effect profile in the form of lesser respiratory depression, nausea, and vomiting.[23] It can be safely used in clinically significant hepatic or renal diseases.[24] During MAC, this drug can be used as intermittent bolus doses or as a continuous IV infusion as total intravenous anesthesia (TIVA) or as a combination of the two. However, all techniques of MAC require active patient monitoring during and after the procedure for the potential adverse effects of opioid usage, especially respiratory depression, postoperative nausea, vomiting, and dizziness.

The avoidance of a general anesthetic during ESWL is advantageous reducing the morbidity and potential mortality and allowing treatment on an outpatient basis, indirectly reducing cost. However, the use of general anesthetic results in more controlled respiratory excursion, which translates into more effective stone targeting and fragmentation. Therefore, general anesthesia may be preferred in following conditions - children, extremely anxious individuals, when a lengthy treatment is anticipated, e.g., bilateral ESWL, concomitant renal and ureteral stones. Calculi composed of cystine, calcium oxalate monohydrate or brushite are known to be resistant to fragmentation. Therefore, if their presence is anticipated, delivery of higher levels of shockwave energy with attendant increased anesthesia requirements should be expected. Thin patients have more pain during ESWL because the converging shockwave is more concentrated at the point of skin penetration.

Regional anesthesia has utilized intrathecal lidocaine and sufentanil during ESWL.[25–28] These techniques are, however, more time consuming to perform and the results in prolonged recovery due to residual sympathetic blockade. intrathecal sufentanil is a safer and an effective alternative to lidocaine, resulting in early ambulation and discharge, ability to void, most likely due to preservation of motor and sensory function.[27] However, its use results in undesirable pruritis in addition to requiring active patient monitoring.[25,27]

More recently, prilocaine has been used in the form of subcutaneous infiltration during ESWL.[29] This drug temporarily inhibits nerve fiber conduction by blocking transfer of sodium ions into the cell across the cell membrane. In comparison to lidocaine, it has a rapid onset of action, equal efficacy, and duration of effect but with lesser toxic effects due to rapid metabolism. This drug when given 1-2 min before the procedure in the target area resulted in better pain control with lesser supplementary analgesia requirement than intramuscular (IM) diclofenac.[29] Its main drawback includes injection of the drug at the precise site of skin entry of shockwaves and larger studies are needed to validate this finding.

Patient-controlled analgesia has been introduced to avoid the problems of bolus doses of IV opioids, such as respiratory depression and delayed discharge.[30] A small dose is delivered via an IV pump when the patient presses a button. The pump has a built in safety mechanism, so that the next dose cannot be given for a set period. It has been suggested that patient-controlled analgesia enables urologists to achieve better compliance through more accurate pain control and hence more effective treatment.[31] Hence, patient can be given the option of a patient-controlled analgesia pump during treatment with ESWL, especially when presenting for a second or subsequent treatment, so that they may know what level of pain to expect. However, this technique requires an intelligent patient along with an expensive device, which is not universally available and there can be problems of malfunctioning of device. Therefore, this technique is still not universally acceptable.

NSAIDS like diclofenac sodium provide pain relief by their anti-inflammatory effect caused by prostaglandins synthesis inhibition and are effective via oral, IM, and rectal routes. It is an effective analgesic with lower side effects than opioids especially with regard to hemodynamic instability and respiratory depression.[20] However, it is associated with mild gastrointestinal disturbances, occasional hypersensitivity reactions, and sometimes coagulation disorders because of cyclo-oxygenase inhibition.[32]

The EMLA cream, a eutectic mixture of lignocaine (2.5%) and prilocaine (2.5%) for topical use, has also been used in ESWL as an occlusive dressing due to its local anesthetic effect and its action like a coupling medium.[33] It can penetrate to a depth of 4 mm through intact skin after 60 mins of application.[34,35] Its use as a topical anesthetic for venous catheterization, condyloma acuminatum excision, and preparation of skin grafts, has been reported in various series.[34,35] Though some reports have found EMLA cream to be an ineffective analgesic agent without any opioid-sparing effect,[36] others have found it to be a good alternative to other analgesics because of its simplicity and noninvasiveness, avoiding the side effects of IM or IV analgesic agents.[7,9] It reportedly reduces opioid requirement by 23% during ESWL performed with newer lithotriptors, thus reducing their side effects.[37] However, it should be emphasized that it should be used appropriately as an occlusive dressing in combination with other analgesic agents like opioids because its own analgesic effect is inefficient.[9] Moreover, this cream has to be applied 45-60 min before the procedure to achieve its maximum effect.[38] Interestingly, most studies evaluating EMLA cream during ESWL did not use it as an occlusive dressing. This may have been the reason for their unfavorable results with pain control, as effectiveness of occlusive dressing enhances the local anesthetic effect of EMLA, due to absorption of shock waves, thus allowing maximum energy to reach the focal point.[33]

We performed a prospective randomized study comparing the efficacy of oral diclofenac, occlusive dressing of EMLA cream, and their combination separately.[38] Efficacious analgesia was achieved during the procedure, with minimal side effects, avoiding the need for an injectable analgesic in patients receiving the combination of oral diclofenac and occlusive dressing of EMLA cream. Moreover, the success rate of the procedure was also increased. The combination can be used safely in hypertensive and diabetic patients. However, NSAIDS should not be used in patients with renal impairment.

Recently, the use of dimethyl sulfoxide (DMSO) in combination with lidocaine has been reported to provide better pain control during ESWL as compared to EMLA cream, due to local anesthetic effect along with diuretic, anti-inflammatory, muscle relaxant, and hydroxyl radical scavenger effects of DMSO.[39–41] However, large scale randomized controlled trials are required for validating its use.

## CONCLUSIONS

Despite the introduction of new generation lithotriptors, there is need for effective analgesia during ESWL for optimal stone fragmentation. The ideal analgesia, which offers pain-free treatment, minimal side effects, and adequate cost-effectiveness, remains to be established. Although, opioids provide effective analgesia, they require active monitoring of patient for various potential adverse effects. Combination therapy (oral NSAID and occlusive dressing of EMLA, DMSO with lidocaine) offers an effective alternative mode for achieving analgesia with minimal morbidity. This therapy avoids the need for general anesthesia, injectable analgesics, and opioids along with their side effects

## References

[1] Chaussy C, Brendel W, Schmiedt E (1980). Extracorporeal induced destruction of kidney stones by shock waves. Lancet.

[2] Chaussy GC, Fuchs GJ (1989). Current state and future developments of noninvasive treatment of urinary stones with extracorporeal shock wave lithotripsy. J Urol.

[3] Salinas AS, Lorenzo-Romero J, Segura M, Calero MR, Hernandez-Millan I, Martinez-Martin M (1999). Factors determining analgesic and sedative drug requirements during extracorporeal shock wave lithotripsy. Urol Int.

[4] Wickham JE (1993). Treatment of urinary tract stones. BMJ.

[5] Hosking MP, Morris SA, Klein FA, Dobmeyer-Dittrich C (1997). Anesthetic management of patients receiving calculus therapy with a third-generation extracorporeal lithotripsy machine. J Endourol.

[6] Schelling G, Weber W, Mendl G, Braun H, Cullmann H (1996). Patient controlled analgesia for shock wave lithotripsy: The effect of self administered alfentanil on pain intensity and drug requirement. J Urol.

[7] Yilmaz E, Batislam E, Basar MM, Tuglu D, Ozcan S, Basar H (2005). Effectiveness of eutectic mixture of local anesthetic cream and occlusive dressing with low dosage of fentanyl for pain control during shock wave lithotripsy. J Endourol.

[8] Gesztesi Z, Sa Rego M, White F (2000). The comparative effectiveness of fentanyl and its newer analogs during extracorporeal shock wave lithotripsy under monitored anesthesia care. Anesth Analg.

[9] Basar H, Yilmaz E, Ozcan S, Buyukkocak U, Sari F, Apan A (2003). Four analgesic techniques for shock wave lithotripsy: Eutectic mixture local anesthetic is a good alternative. J Endourol.

[10] Parkin J, Keeley FX, Timoney AG (2002). Analgesia for shock wave lithotripsy. J Urol.

[11] Chaussy C, Schmiedt E, Jocham D (1982). First clinical experience with extracorporeally induced destruction of kidney stones by shock waves. J Urol.

[12] Clayman R, McClennan B, Garvin T (1989). Lithostar: An electromagnetic acoustic unit for extracorporeal lithotripsy. J Endourol.

[13] Rassweiler J, Kohrmann KU, Junemann KP, Alken P, Smith AD (1995). Use of electromagnetic technology. Chapter 5. Types of Extracorporeal lithotriptors. Controversies in Endourology.

[14] Chow GK, Streem SB (2000). Extracorporeal lithotripsy-update on technology. Urol Clin North Am.

[15] Knudsen F, Jorgensen S, Bonde J, Andersen JT, Mogensen P (1992). Anesthesia and complication of extracorporeal shock wave lithotripsy of urinary calculi. J Urol.

[16] Tritrakarn T, Lertakyamanee J, Tuntiwong A, Raksamani A, Jittrapapal S (1992). Evaluation of pain during extracorporeal piezoelectric lithotripsy with Piezolith 2300. Thai J Anesthesiol.

[17] Weber A, Koehrmann KU, Denig N, Michel MS, Alken P (1998). What are the parameters for predictive selection of patients requiring anesthesia for extracorporeal shock wave lithotripsy?. Eur Urol.

[18] Vickers MD, Morgan M, Sencer PS (1991). Drugs in anesthetic practice. Systemic analgesics.

[19] Monk TG, Boure B, White PF, Meretyk S, Clayman RV (1991). Comparison of intravenous sedative-analgesic techniques for outpatient immersion lithotripsy. Anesth Analg.

[20] Cohen E, Hafner R, Rotenberg Z, Padilla M, Garty M (1998). Comparison of ketorolac and diclofenac in the treatment of renal colic. Eur J Clin Pharmacol.

[21] Cortinez LI, Munoz HR, De la Fuente R, Acuna D, Dagnino JA (2005). Target-controlled infusion of remifentanil or fentanyl during extra-corporeal shock-wave lithotripsy. Eur J Anaesthesiol.

[22] Medina HJ, Galvin EM, Dirckx M, Banwarie P, Ubben JF, Zijlstra FJ (2005). Remifentanil as a single drug for extracorporeal shock wave lithotripsy: A comparison of infusion doses in terms of analgesic potency and side effects. Anesth Analg.

[23] Beloeil H, Corsia G, Coriat P, Riou B (2002). Remifentanil compared with sufentanil during extra-corporeal shock wave lithotripsy with spontaneous ventilation: A double-blind, randomized study. Br J Anaesth.

[24] Sa Rego MM, Inagaki Y, White PF (1999). Remifentanil administration during monitored anesthesia care: Are intermittent boluses an effective alternative to a continuous infusion?. Anesth Analg.

[25] Eaton MP, Chhibber AK, Green DR (1997). Subarachnoid sufentanil versus lidocaine spinal anesthesia for extracorporeal shock wave lithotripsy. Reg Anesth.

[26] Lau WC, Green CR, Faerber GJ, Tait AR, Golembiewski JA (1999). Determination of the effective therapeutic dose of intrathecal sufentanil for extracorporeal shock wave lithotripsy. Anesth Analg.

[27] Lau WC, Green CR, Faerber GJ, Tait AR, Golembiewski JA (1997). Intrathecal sufentanil for extracorporeal shock wave lithotripsy provides earlier discharge of the outpatient than intrathecal lidocaine. Anesth Analg.

[28] Kanazi GE, Tran SB, Rizk L, Baraka A (2003). Multimodal spinal anesthesia. Middle East J Anesthesiol.

[29] Yilmaz E, Batislam E, Basar M, Tuglu D, Yuvanc E (2006). Can prilocaine infiltration alone be the most minimally invasive approach in terms of anesthesia during extracorporeal shock wave lithotripsy?. Urology.

[30] White PF (1988). Use of patient controlled analgesia for management of acute pain. JAMA.

[31] Chin CM, Tay KP, Ng FC (1997). Use of patient controlled analgesia in extracorporeal shock wave lithotripsy. Br J Urol.

[32] Power I, Chambers WA, Greer IA, Ramage D, Simon E (1990). Platelet function after intramuscular diclofenac. Anesthesia.

[33] Tritrakarn T, Lertakyamanee J, Koompong P, Soontrapa S, Somprakit S, Tantiwong A (2000). Both EMLA and placebo cream reduced pain during extracorporeal piezoelectric shock wave lithotripsy with the piezolith 2300. Anesthesiology.

[34] Xavier B, Caffaratti J, Orsola A, Garat JM, Vicente GJ (1996). Topical anesthesia with the EMLA cream: Application in pediatric urology. Actas Urol Esp.

[35] Aldret-Neilson L, Bjerring P, Nielson J (1999). Regional variations in analgesic efficacy of EMLA cream. Acta Dermatol Scand.

[36] Ganapathy S, Razoi H, Moote C, Parkin J, Yee I, Gverzdys S (1996). Eutectic mixture of local anesthetics is not effective for extracorporeal shock wave lithotripsy. Can J Anesth.

[37] Monk TG, Ding Y, White PF, Albala DM, Clayman RV (1994). Effect of topical eutectic mixture of local anesthetics on pain response and analgesic requirement during lithotripsy procedures. Anesth Analg.

[38] Kumar A, Gupta NP, Hemal AK, Wadhwa P (2007). Comparison of three analgesic protocols for pain control during shock wave lithotripsy using dornier delta compact lithotripter: A prospective randomized clinical trial. J Endourol.

[39] Demir E, Kilciler M, Bedir S, Erten K, Ozgok Y (2007). Comparing two local anesthesia techniques for extracorporeal shock wave lithotripsy. Urology.

[40] Birkmayer W, Danielezyk W, Werner H, Laudahn G, Gertich K (1966). DMSO ber spondylogenen neuropathien. DMSO symposium.

[41] Panganamala RV, Sharma HM, Heikkila RF (1976). Role of hydroxyl radical scavengers, dimethylsulfoxide, alcohols and methional in the inhibition of prostaglandin synthesis. Prostaglandins.

